# Reduced prefrontal cortex and sympathetic nervous system activity correlate with fatigue after aHSCT

**DOI:** 10.1038/s41409-021-01539-9

**Published:** 2021-12-04

**Authors:** Erik Boberg, Ellen Iacobaeus, Myrto Sklivanioti Greenfield, Yanlu Wang, Mussie Msghina, Katarina Le Blanc

**Affiliations:** 1 Department of Laboratory Medicine, Karolinska Institutet, Stockholm, Sweden; 2 Department of Haematology, Karolinska University Hospital, Stockholm, Sweden; 3 Department of Clinical Neuroscience, Karolinska Institutet, Stockholm, Sweden; 4 Medical Radiation Physics and Nuclear Medicine, Karolinska University Hospital, Stockholm, Sweden; 5 Radiology, Clinical Sciences, Intervention and Technology, Karolinska Institutet, Stockholm, Sweden; 6 School of Medical Sciences, Örebro University, Örebro, Sweden; 7 Department of Cellular therapy and Allogeneic Stem Cell Transplantation, Karolinska University Hospital, Stockholm, Sweden

**Keywords:** Medical imaging, Quality of life

## Abstract

Long-term fatigue and cognitive dysfunction affects 35% of allogeneic haematopoietic stem cell transplantation (aHSCT) survivors, suggesting a dysfunctional prefrontal cortex. In this study, we assessed prefrontal cortex and sympathetic nervous system activity in aHSCT patients with fatigue (*n* = 12), non-fatigued patients (*n* = 12) and healthy controls (*n* = 27). Measurement of near-infrared spectroscopy and electrodermal activity was carried out at rest and during cognitive performance (Stroop, verbal fluency and emotion regulation tasks). Prefrontal cortex and sympathetic nervous system activity were also analyzed in response to dopamine and noradrenaline increase after a single dose of methylphenidate. Baseline cognitive performance was similar in the two patient groups. However, after methylphenidate, only non-fatigued patients improved in Stroop accuracy and had better verbal fluency task performance compared to the fatigued group. Task-related activation of prefrontal cortex in fatigued patients was lower compared to non-fatigued patients during all cognitive tests, both before and after methylphenidate administration. During the Stroop task, reaction time, prefrontal cortex activation, and sympathetic nervous system activity were all lower in fatigued patients compared to healthy controls, but similar in non-fatigued patients and healthy controls.

Reduced prefrontal cortex activity and sympathetic arousal suggests novel treatment targets to improve fatigue after aHSCT.

## Introduction

Improved patient care has increased survivorship after allogeneic haematopoietic stem cell transplantation (aHSCT), but sequelae remain common [1]. Persisting fatigue and cognitive dysfunction, such as executive dysfunction, memory loss, difficulties in concentration and completing tasks are commonly reported in cancer treated patients, including aHSCT recipients [2–4]. These impairments negatively impact the patients’ quality of life and ability to return to employment. Although highly clinically relevant, the underlying pathophysiology associated with fatigue and cognitive dysfunction remains elusive [3, 5, 6].

Central nervous system (CNS) complications from cancer regimens are well known. Cognitive dysfunction is among the most common manifestations [7]. However, it is unknown if the allogenic transplantation itself directly impacts on processes involved in higher cortical functions. Alterations in gray matter volumes and white matter connectivity one year after aHSCT have been reported, suggesting that the transplant procedure affects CNS structures [8, 9].

Executive functions are commonly perturbed in both solid tumor and aHSCT survivors [5, 10–12]. These constitute cortical processes utilized in complex or novel situations where the individual cannot rely on instinct or automatic behavior [13]. Traditionally, executive functions have been attributed to the prefrontal cortex [14]. Prefrontal cortex function is highly dependent on the neurotransmitters dopamine and noradrenaline, both implicated in the cognitive dysfunction seen in Parkinson’s disease, schizophrenia, and attention deficit hyperactivity disorder [15–19]. Both dopamine and noradrenaline exhibit an inverted U-formed relationship between activity and performance, meaning that moderate concentrations are optimal, while both high and low concentrations are detrimental to prefrontal cortex function [18, 20]. Clinical trials of methylphenidate treatment of fatigue or cognitive dysfunction in cancer patients have been inconclusive, suggesting that the dopaminergic or noradrenergic systems are unresponsive to this medication [21–24].

Functional near-infrared spectroscopy (fNIRS) detects regional brain activity by measuring shifts in blood oxygenation when metabolic demand increases during cortical activation [25]. An array of photodiode detectors record emitted near-infrared light refracted by the cortical brain tissue [26]. fNIRS data processing is similar to functional MRI (fMRI) data, and the results are normally directly comparable [27]. fNIRS is limited in brain coverage and spatial resolution, but offers better temporal resolution, lower cost and greater mobility compared to fMRI.

Greater task-related regional brain activity may not necessarily imply better functioning. Instead, reduced cognitive function due to aging or injury can be compensated by wide-spread activity, or by over-activating different cortical regions [28–30]. Thus, assessment of cortical activity must take the subject’s cognitive performance into account.

Performing cognitively demanding tasks result in heightened arousal which possibly contributes to optimization of task performance [31–33]. The sympathetic nervous system innervates sweat glands and increased sweat production causes increased skin conductance [34]. Electrodermal activity (EDA) can be measured with skin electrodes. Previous studies suggest that cancer patients with fatigue have impaired sympathetic nervous system function [35, 36].

We recently reported that long-term aHSCT survivors with persistent fatigue suffer from impaired executive functions [37]. The primary aim of this study was to investigate if this executive impairment is due to prefrontal cortex dysfunction. We compared prefrontal cortex activity in fatigued and non-fatigued aHSCT recipients during cognitive task performance using fNIRS. Secondarily, we hypothesized that the prefrontal cortex of fatigued and non-fatigued patients would respond differently to increased dopamine and noradrenaline concentrations, and therefore repeated all measurements after a single-dose of methylphenidate [38]. Finally, sympathetic nervous system activity was recorded under the hypothesis that reduced sympathetic arousal may contribute to the lower cognitive performance seen in fatigued patients.

## Materials and methods

### Study subjects

This study was performed on a subset of patients previously recruited [37]. Patients 1–5 years post aHSCT were classified as fatigued (*n* = 12) or non-fatigued (*n* = 12) based on self-reported symptoms and mental fatigue scale score (MFS [39], ≥14 = fatigued, ≤10 = non-fatigued). Patients were ≥18, spoke fluent Swedish, and had no history of neurologic or severe psychiatric disorder. We excluded patients treated with intrathecal chemotherapy, CNS irradiation, total body irradiation or significant doses of psychoactive drugs. Brain MRI excluded neurological diseases and significant structural abnormalities. For further details, refer to Supplementary Table 1 and the original publication [37]. We excluded patients from the cohort that had contraindications to methylphenidate treatment (Supplementary Table 2). All participants provided written consent. The study was conducted in agreement with the declaration of Helsinki and approved by the regional ethics committee in Stockholm, Sweden.

### Healthy control data

**Fig. 1:**
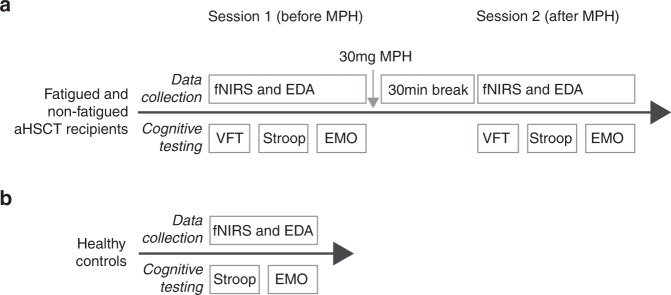
Experiment procedure. **A** The fatigued and non-fatigued patients underwent three cognitive tests, the VFT, Stroop and negative emotion regulation tasks. During the testing, fNIRS and EDA registration was performed. After the testing, all patients received a single dose of 30 mg methylphenidate and a 30 min break was given to allow drug absorption. No food or drinks were allowed during the break, except for water. After the break, the same testing procedure was repeated. **B** The HC only underwent one session of testing, without any medication. VFT Verbal Fluency Task, fNIRS functional Near-infrared Spectroscopy, EDA Electrodermal Activity, methylphenidate Methylphenidate, HC Healthy Controls.

The measurements conducted in this study were compared to data from a previous study from our group (Sklivanioti et al., manuscript in preparation) that analyzed healthy subjects using the same technical equipment and identical protocols as the current study (Fig. 1B). We included 27 healthy controls from that study that had completed the emotion regulation task. Among those, 25 subjects had also completed the Stroop task.

### Data acquisition protocol

All subjects were right-handed as assessed by the Edinburgh Handedness Inventory – short form [40] prior to testing. All other measurements were carried out in a dimly lit and sound isolated room during two identical sessions, each lasting approximately an hour. fNIRS and EDA measurements were conducted while the participants performed three cognitive tasks: the verbal fluency task (VFT), Stroop task and an emotion regulation task. The tasks were presented on a computer screen, and data was recorded using Eprime software version 2.1 (Psychology software tools, Sharpsburg, PA, USA). Immediately after session one, all subjects were given a single oral dose of 30 mg short acting methylphenidate (Ritalin©), administrated single blinded to the patients. A 30-minute break allowed for medication uptake. Food or beverages other than water were disallowed. Fatigue level was measured using a numeric rating scale (NRS) before and after each session. The protocol is summarized in Fig. 1.

### Cognitive tests

The Stroop task tests cognitive control and resolving cognitive interference [41]. The test was divided into 8, 30-s-long blocks of 15 trials per block, randomized between congruent and incongruent tasks, with 30 s rest in between. Median reaction time and proportion of correct answers were registered [42]. Unanswered trials and trials with a reaction time <200 ms were removed [42]. Before registration, the subjects could practice until they felt comfortable with the paradigm. During the VFT, five vowels were presented in sequence. The subjects were instructed to generate as many words as they could, beginning with each vowel, within 20 s. The test started with a rest phase followed by a vocalization phase.

The emotion regulation task consisted of 6 blocks with 30 s of rest in between, during which five images with negative emotional valence were presented in sequence [43]. The subjects were instructed to either observe passively (emotion induction) or actively attempt to reduce the emotion intensity evoked by the images (emotion regulation). The outcome measure was the relative difference in emotion rating during the emotion induction and regulation tasks. For details, see Supplementary Methods.

### Data acquisition

fNIRS registration was performed using a Biopac fNIR model 1100-V3.2A (fNIR Devices LLC, Potomac, MD, USA) with a 16-channel sensor placed over the subject’s forehead. After converting the raw data to relative changes in oxygenated and deoxygenated hemoglobin using the modified Beer-Lambert equation, activation maps corresponding to the cognitive tasks was constructed using generalized linear models. Finally, channel-wise comparisons were made between the study groups. The EDA signal was recorded using the MP150 data acquisition and analysis system (Biopac systems inc, Goleta, CA, USA) through silver-silver chloride electrodes attached to dig 2 and 3 of the left hand (Supplementary Fig. 1e). The EDA signal was decomposed into three features that were analyzed in this study; (i) the increase in electrodermal level between rest and task performance, (ii) the mean non-specific electrodermal response (EDR) amplitude (both reflecting overall degree of arousal), and (iii) the mean task-related EDR amplitude (reflecting short term responses to external stimuli) [44, 45]. For details, see Supplementary Methods.

### Statistical analysis

For paradigm performance and NRS, normality was tested with the Shapiro Wilks test. T-tests or Wilcoxon rank-sum tests were selected as appropriate. fNIRS channel beta-coefficients were analyzed as follows: Activation/deactivation was determined with one-sample t-tests and between/within group differences using unpaired/paired t-tests. For activated/deactivated channels, Pearson correlations of between-session activity changes and performance changes were calculated. EDA data was analyzed with linear mixed effects modeling. The patient data acquired before and after methylphenidate were separately compared to the healthy control (HC) data. Due to age differences between patients and HC, all comparisons to HC were age-adjusted. For details, see Supplementary Methods.

## Results

Of the 27 participants in the original cohort, 24 (12 fatigued and 12 non-fatigued) were included in this study [37]. There were no significant differences between the patient groups with regards to disease- or transplant-related characteristics (Table 1). The patient groups were older compared to HC (Supplementary Table 3).

**Table 1:** Characteristics of the study population.

	Fatigued (*n* = 12)	Non-fatigued (*n* = 12)	*P*
Time from transplant to fNIRS testing (months): median (range)	46 (16–66)	47.5 (22–68)	0.68^e^
Donor type: *n* (%)			
MUD	1 (8)	1 (8)	1^g^
SIB	4 (33)	4 (33)	
Haplo	7 (58)	7 (58)	
Underlying disease: *n* (%)			
AML	4 (33)	9 (75)	0.11^g^
CML	0 (0)	0 (0)	
MDS	1 (8)	2 (17)	
MDS/AML	1 (8)	0 (0)	
PMF	3 (25)	0 (0)	
PMF/AML	0 (0)	1 (8)	
Myeloma	1 (8)	0 (0)	
CLL	1 (8)	0 (0)	
Sickle cell anemia	1 (8)	0 (0)	
Conditioning regimen drugs: *n* (%)			
Busulfan	8 (67)	9 (75)	1^g^
Cyclophosphamide	3 (25)	2 (17)	1^g^
Fludarabine	9 (75)	10 (83)	1^g^
Treosulfan	4 (33)	3 (25)	1^g^
Thiotepa	2 (17)	0 (0)	0.48^g^
Immune reconstitution			
Days from transplant to neutrophils > 0.5 × 10^9^/L (days): median (range)	16 (12–28)	17 (11–19)	0.93^f^
CMV and EBV: *n* (%)			
CMV mismatch	4 (33)	2 (17)	0.64^g^
EBV mismatch	0 (0)	1 (8)	1^g^
CMV reactivation^a^	6 (50)	4 (33)	0.68^g^
EBV reactivation^b^	2 (17)	1 (8)	1^g^
GvHD prophylaxis: *n* (%)			
ATG	8 (67)	7 (58)	1^g^
Ciclosporin	8 (66)	9 (75)	0.68^g^
Tacrolimus	3 (25)	1 (8)	
Tacrolimus + Sirolimus	1 (8)	2 (17)	
GvHD: *n* (%)			
Acute^c^	9 (75)	7 (58)	0.67^g^
Chronic^d^	5 (42)	4 (33)	1^g^
Regular opioid, antidepressive or anxiolytic medication: *n* (%)			
SSRI	2 (17)	0 (0)	0.48^g^
Opioids	2 (17)	0 (0)	0.48^g^

*MUD* matched unrelated donor, *SIB* matched sibling donor, *Haplo* haploidentical donor, *AML* acute myeloid leukemia, *CML* chronic myeliod leukemia, *MDS* myelodysplastic syndrome, *PMF* primary myelofibrosis, *CLL* chronic lymethylphenidateocytic leukemia, *CMV* cytomegalovirus, *EBV* Epstein Barr virus, *GvHD* graft versus host disease, *ATG* anti thymocyte globulin, *SSRI* selective serotonin reuptake inhibitor.

^a^Defined as CMV DNA > 1000 copies/ml.

^b^Defined as EBV DNA > 1000 copies/ml.

^c^Previous acute GvHD requiring steroid treatment.

^d^Previous/current cGvHD.

^e^Students *t* test (continuous data with parametric distribution according to the Shapiro–Wilk test).

^f^Wilcoxon Rank Sum Test (continuous data with non-parametric distribution according to the Shapiro–Wilk test).

^g^Fisher’s exact test (categorical data).

Fatigue intensity was measured with the NRS at the start and end of each session (before and after methylphenidate). Fatigue patients rated fatigue intensity higher at all timepoints compared to non-fatigue patients, and their fatigue intensity increased significantly during the first but not during the second session. In non-fatigue group, fatigue intensity remained low and constant (Supplementary Fig. 2).

### Baseline cognitive task performance was similar in the patient groups, but prefrontal cortex activity was lower in the fatigued compared to the non-fatigued patients

**Fig. 2:**
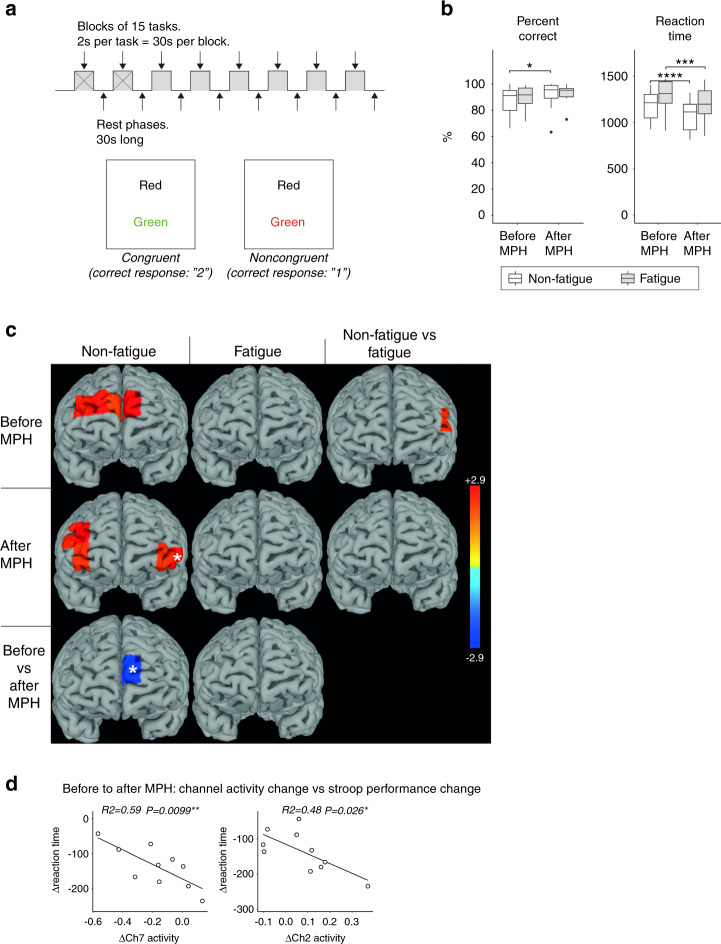
The Stroop task - no improvement in accuracy after methylphenidate and no significant task-related prefrontal cortex activity in fatigued patients. **A** The Stroop task was divided into 8 blocks of 15 tasks per block. Examples of a congruent and noncongruent trial are provided. **B** Stroop task performance was similar between fatigued and non-fatigued patients before and after methylphenidate. Both groups improved in reaction time after receiving methylphenidate, but only the non-fatigued group significantly improved in accuracy (% correct answers). **C** The heatmaps display t-values for the significant comparisons between task and rest, non-fatigued and fatigued as well as before and after methylphenidate. Before methylphenidate, the non-fatigued patients activated medially located channels 7, 9, 11 and 13 while the fatigued patients showed no activation. Channel 1 was significantly more active in non-fatigued compared to fatigued patients. After methylphenidate, the non-fatigued patients deactivated the medially located channel 7 and significantly activated bilaterally located channels 2, 4, 13, 14 and 15, while the fatigued patients still showed no significant task-related activity. **D** Correlation analyses were performed between channel activity difference and Stroop performance difference between the two sessions. Only channels that were significantly activated or deactivated after methylphenidate, or with significant difference in activity from before to after methylphenidate were included. Compared to before methylphenidate administration, a greater increase in channel 2 and 7 activity was correlated to a more reduced reaction time in the non-fatigued group. The channels for which the correlations were significant are labeled with white stars in **C**. methylphenidate Methylphenidate, Ch Channel.

**Fig. 3:**
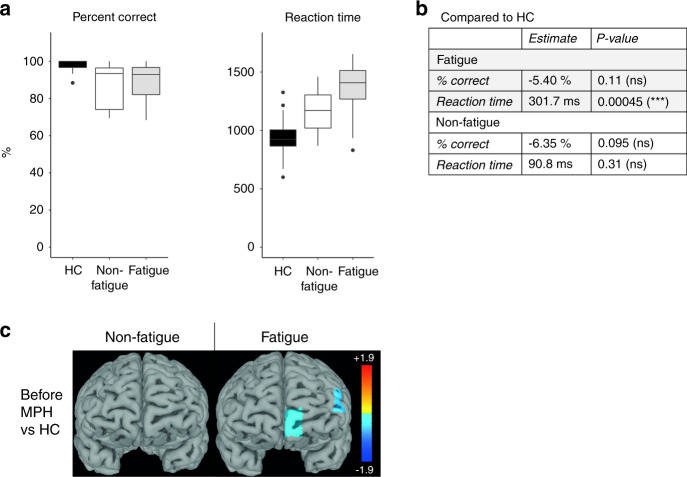
The Stroop task – significantly lower performance and prefrontal cortex activation in fatigued patients compared to healthy controls. Since the HC only completed 4 blocks of the Stroop task, the HC data was compared to the first 4 blocks during session 1. To obtain age-adjusted comparisons, the Stroop reaction time and percent correct answers were compared between the fatigued/non-fatigued groups and HC using linear models with age as independent variable. Only data from session 1 was used. **A**, **B** Both the fatigued and non-fatigued groups were equally correct compared to HC, but the fatigued group had significantly slower reaction time compared to the HC, while the non-fatigued group had similar reaction times as the HC after adjusting for age. **C** The heatmaps display t-values for the significant comparisons between fatigued/non-fatigued groups and the HC group before methylphenidate. Compared to HC the fatigued patients had significantly lower activation in channels 1 and 8. The non-fatigued patients were not different from HC. HC Healthy Controls, methylphenidate Methylphenidate.

Baseline Stroop performance was similar in both patient groups (Fig. 2B). Compared to unmedicated HC, both patient groups had similar rates of accuracy after adjusting for age (Fig. 3B). However, age-adjusted reaction time was significantly slower only in the fatigued group compared to HC (*p* = 0.00045). The Stroop task did not activate any fNIRS channels compared to rest in fatigue patients. In contrast, non-fatigue patients activated dorsally located channels (channels 7, 9, 11 and 13), and a dorsolateral channel (channel 1) was significantly more active compared to the fatigue group (Fig. 2C). Age-adjusted task-related activation in the prefrontal cortex was similar in non-fatigue patients and HC. In contrast, fatigue patients had significantly lower activity in left dorsolateral and ventromedial channels 1 and 8 (Fig. 3C).

**Fig. 4:**
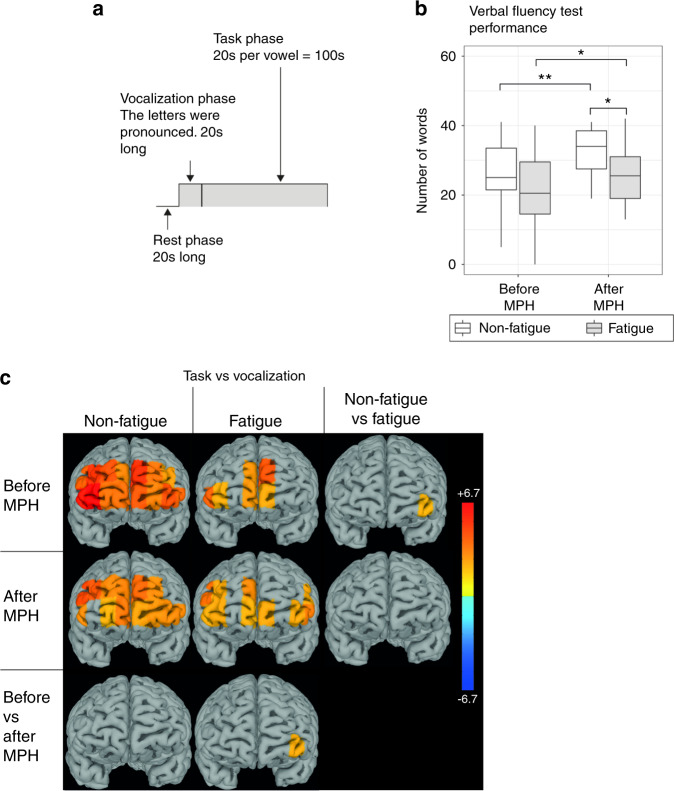
The VFT – more pronounced improvement and more channels activated in non-fatigued compared to fatigued. **A** The VFT was divided in three phases: A 20 s rest phase, followed by a 20 s vocalization phase and finally the task, which was 100 s long. The HbO levels was compared between the task and vocalization phases. **B** There was no significant difference in number of words produced between fatigued and non-fatigued before methylphenidate. Both groups improved after methylphenidate administration, but the non-fatigued improved to a greater extent, resulting in significantly more words produced, compared to fatigued. **C** The heatmaps display t-values for the significant comparisons between task and rest, non-fatigued and fatigued as well as before and after methylphenidate. Significant increase in prefrontal cortex activity from vocalization to task was seen in 15/16 channels in non-fatigued and 6/16 channels in fatigued patients before methylphenidate. After methylphenidate the non-fatigued activated 13 channels and fatigued activated 10 channels. Channel 4 was significantly more activated in non-fatigued compared to fatigued patients before methylphenidate, and significantly increased in activity in fatigued patients after methylphenidate administration. The heatmaps display t-values for the comparisons between task and rest, non-fatigued and fatigued as well as before and after methylphenidate. VFT Verbal Fluency Task, HbO Oxygenated Hemoglobin, methylphenidate Methylphenidate.

During VFT, both patient groups produced a similar number of words at baseline (Fig. 4B). In non-fatigued patients, VFT caused widespread activation in the prefrontal cortex, including all channels but channel 1. In contrast, fatigue patients significantly activated only 6 of the 16 channels (7, 8, 9, 10, 14 and 16, Fig. 4C). Left ventrolateral PFC (channel 4) was significantly more active in non-fatigue compared to fatigue patients.

**Fig. 5:**
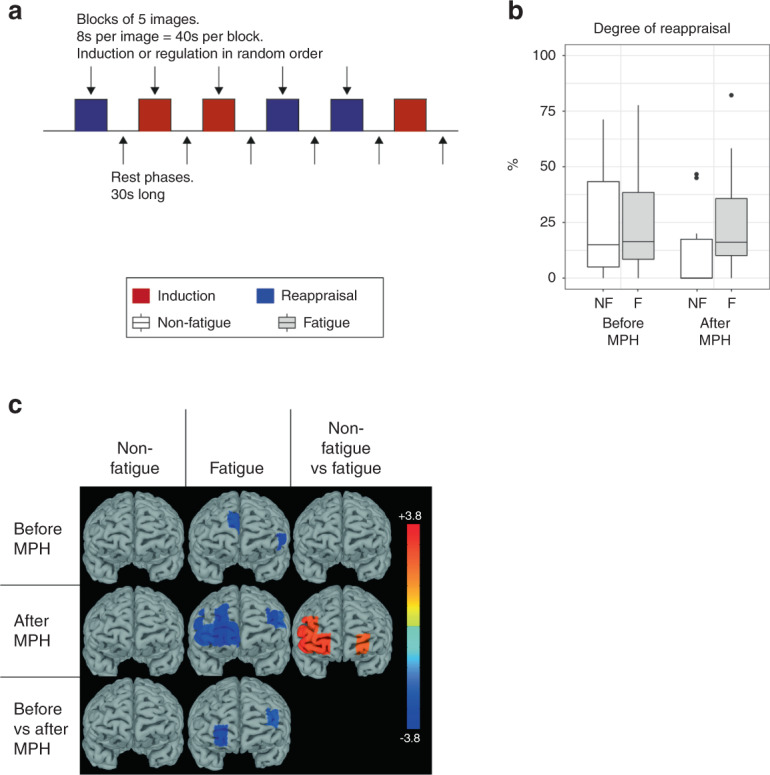
Emotion regulation – lower prefrontal cortex activity in fatigued compared to non-fatigued patients. **A** The paradigm consisted of 6 blocks of 5 images of negative emotional valence shown in sequence. The subjects were instructed in randomized order to either passively view the images (induction) or to attempt to regulate the negative emotions that the images evoked through reappraisal. **B** The degree of emotional regulation was similar between the groups before and after methylphenidate. **C** The heatmaps display t-values for the significant comparisons between task and rest, non-fatigued and fatigued as well as before and after methylphenidate. Despite similar behavioral results, the prefrontal cortex activity differed between the groups, where a deactivation of 2 channels before methylphenidate and 10 channels after methylphenidate was seen for the fatigued group, but no channels for the non-fatigued group. This resulted in higher activity in 6 channels in the non-fatigued compared to the fatigued group. methylphenidate: Methylphenidate.

**Fig. 6:**
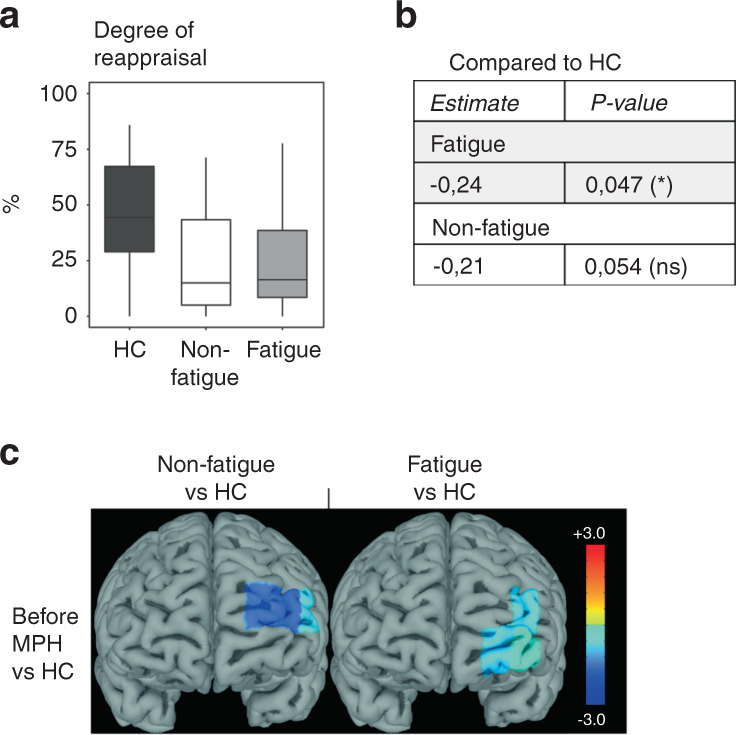
The negative emotion task – lower degree of suppression despite increased prefrontal cortex activity after methylphenidate in both patient groups compared to HC. **A** The degree of regulation was lower in both fatigued and non-fatigued groups compared to HC. The age-adjusted *p* values for the comparisons to HC are noted in **B**. **C** The heatmaps display prefrontal cortex activity (t-values) for the significant comparisons between fatigued/non-fatigued groups and the HC group before methylphenidate. The lower degree of emotional regulation compared to HC was associated with lower left-sided prefrontal cortex activity in both fatigued and non-fatigued groups. HC Healthy controls, methylphenidate Methylphenidate.

Subjective rating of emotion intensity was lower during emotion regulation compared to emotion induction in both fatigue and non-fatigue patients, resulting in a mean decrease of 28% and 24%, respectively (Fig. 5B). HC reported significantly higher emotion intensity during emotion induction, but similar intensity during emotion regulation, compared to both patient groups, translating to a significantly higher degree of emotion regulation efficiency in HC compared to both patient groups (Fig. 6A, B). Emotion regulation resulted in deactivation of channels 2 and 9 in fatigue patients compared to rest, but not in non-fatigue patients (Fig. 5C). The lower efficiency of emotion regulation in the patient groups compared to HC was associated with a lower degree of left-lateralized prefrontal cortex activity (Fig. 6C).

### Methylphenidate administration affected prefrontal cortex activation differently in fatigued and non-fatigued patients

After methylphenidate, the non-fatigue, but not fatigue patients, improved in Stroop test accuracy (mean ± SEM; non-fatigued: 87.3 ± 3.04% to 91.8 ± 3.40%, *p* = 0.025). Reaction time improved in both patient groups (mean ± SEM; non-fatigued: 1167 ± 52 ms to 1042 ± 54 ms, *p* = 0.0001, fatigued: 1302 ± 70 ms to 1202 ± 63 ms, *p* = 0.00038). Methylphenidate failed to affect prefrontal cortex activity in fatigue patients (Fig. 2C). However, in non-fatigue patients, methylphenidate significantly decreased channel 7 activity and the pattern of activation shifted from dorsomedial to bilateral ventrolateral and dorsolateral channels (channels 2, 4, 13, 14 and 15, Fig. 2C). In non-fatigue patients, changes in activity in channel 2 and 7 after methylphenidate intake correlated with improvement in reaction time (Fig. 2D).

After methylphenidate intake, both patient groups significantly improved their VFT performance (Fig. 4B). However, the improvement in performance was less pronounced in the fatigue group (mean ± SEM; fatigue: 21.2 ± 3.54 to 25.5 ± 2.58 words, *p* = 0.031, non-fatigue: 26.0 ± 2.82 to 32.9 ± 2.02 words, *p* = 0.0026), resulting in significantly lower performance level compared to the non-fatigued group (*p* = 0.035, Fig. 4B). The number of active fNIRS channels decreased slightly from 15 to 13 in non-fatigue patients (channels 2, 4–13, 15 and 16) but increased from 6 to 10 channels in the fatigue group (channels 1, 2, 4, 8–10, 13–16, Fig. 4C). The activity in channel 4 increased significantly in the fatigued group, eliminating the baseline difference between the two groups.

The degree of emotion regulation was not significantly changed after methylphenidate administration. However, fatigue patients increased the number of deactivated channels to 8 (Fig. 5C) while all channels remained inactive in the non-fatigue group, resulting in a higher prefrontal cortex activity in non-fatigue patients compared to the fatigue group.

### Sympathetic nervous system activity was lower in fatigued patients compared to non-fatigued, during the Stroop test

**Fig. 7:**
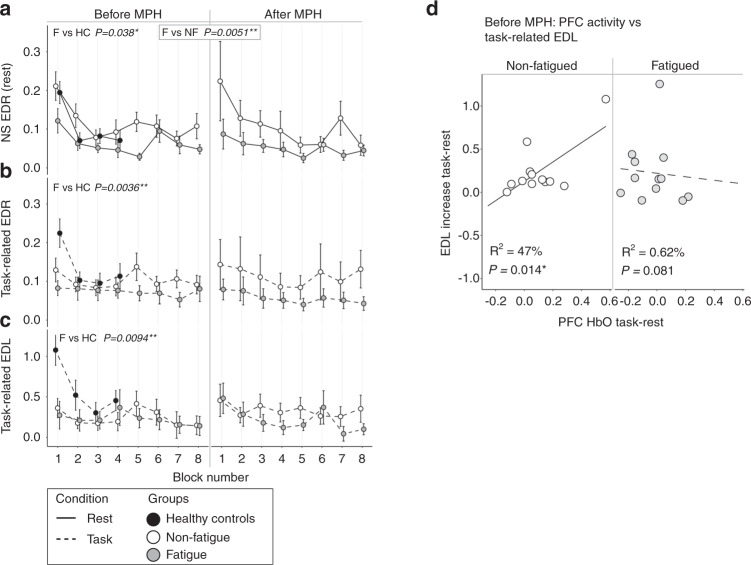
The Stroop task – EDA. The fatigued and non-fatigued groups were compared to each other and to the HC using linear mixed models. The *p* values shown are derived from those models. The comparisons to HC were age-adjusted. Since the HC only completed 4 blocks of the Stroop task, the HC data was compared to the first 4 blocks during session 1. Refer to the materials and methods section for details. **A** The fatigued group had significantly lower NS EDR amplitudes compared to the non-fatigued group, both before and after methylphenidate administration, as well as lower amplitudes compared to HC before methylphenidate. **B**, **C** The task-related EDR amplitudes and task-related EDL increases were significantly lower in the fatigued group compared to HC, before methylphenidate. **D** There was a significant correlation between EDL increase and mean prefrontal cortex activation in the non-fatigued but not the fatigued group, before methylphenidate. HC Healthy controls, EDR Electrodermal Response, NS EDR Non-specific EDR, EDL Electrodermal Level.

During the Stroop test, resting sympathetic nervous system activity was lower in fatigue compared to non-fatigue patients (non-specific EDR: *p* = 0.0051, Fig. 7A). Compared to healthy controls, both resting and task-related sympathetic nervous system activity was lower in fatigue patients (NS EDR: *p* = 0.038; Task-related EDR and EDL: *p* = 0.0036 and *p* = 0.0094 respectively, Fig. 7A–C). Mean prefrontal cortex activity correlated with task-related sympathetic nervous system activity in the non-fatigued but not in the fatigued patients (Fig. 7D). Methylphenidate administration had no effect on resting or task-related sympathetic activity.

During VFT, no difference in EDA was observed between the patient groups and no effect was observed after methylphenidate administration (data not shown).

During emotion regulation, resting sympathetic nervous system activity decreased in fatigued, but not in non-fatigue patients after methylphenidate administration (*p* = 0.026, Supplementary Fig. 3a). Non-fatigue patients had greater baseline sympathetic nervous system activity (task-related EDL increase) compared to HC (*p* = 0.04, Supplementary Fig. 3c).

### Interleukin 8 in cerebrospinal fluid associated with lower prefrontal cortex activity during the Stroop test in the fatigued patients

Since channel 1 activity significantly differed between fatigue and non-fatigue patients, we tested its correlation with cerebrospinal fluid cytokine levels and lymphocyte subset levels analyzed in our previous study [37]. After multiple comparison correction, increasing interleukin 8 (IL-8) levels significantly correlated with reduction of channel 1 activity in the fatigued, but not in non-fatigue patients (*R*^2^ = 76%, *p* = 0.0021, Supplementary Fig. 4).

## Discussion

Cognitive deficiencies after aHSCT are well-known, but their functional neurobiological underpinnings, necessary to develop targeted treatments, have not been investigated. In this study, we found a consistent pattern of hypoactivation of the prefrontal cortex in patients with fatigue during all cognitive tests compared to non-fatigue patients.

The differing prefrontal cortex activation observed between fatigued and non-fatigued patients did not translate to significant differences in baseline cognitive performance. A discrepancy between activation and performance has been described in previous functional brain imaging studies of cancer patients after chemotherapy, and patients with post-traumatic stress disorder [46–50]. These studies used PET or fMRI and were thus able to visualize global cortical activity. Cognitive dysfunction and fatigue in chemotherapy recipients was linked to hyperactivation in some areas and hypoactivation in other areas, compared to HC and chemotherapy-naïve patients [46–49, 51]. The hyperactivation is suggested to be compensatory to impaired cortex function. It is thus possible that our fatigue group compensates frontal hypoactivity by activating less specialized brain regions during cognitive tasks, thereby maintaining performance at the cost of increased energy expenditure and increased fatigue after session 1 as seen in Supplementary Fig. 2. The fact that prefrontal cortex activity of non-fatigue patients was comparable to that of HC during the Stroop task, while the fatigue group showed substantial hypofrontality further supports this theory.

Successful cognitive control of negative emotions has been associated with increased prefrontal cortex and reduced amygdala activation [52, 53]. In our study, the fatigue group rather deactivated the prefrontal cortex in response to the emotion regulation. Deactivation became even more pronounced after methylphenidate intake. It was not observed in the non-fatigue group, yet the efficiency of emotion regulation was comparable between the groups, again suggesting that the fatigue group utilized compensatory cortical areas outside the prefrontal cortex to accomplish the task.

In non-fatigue patients, methylphenidate administration caused a lateralization of prefrontal cortex activation during the Stroop task and a reduction in the number of activated channels during the VFT, paralleled by increased performance. The lateral prefrontal cortex is commonly engaged in the Stroop task and repeated VFT trials have been shown to result in reduction of prefrontal cortex activation [50, 54–57]. Further, in healthy people, methylphenidate intake decreases prefrontal cortex blood flow, while improving cognitive task performance [58]. Thus, the changes in non-fatigue patients after methylphenidate may reflect functional optimization caused by the medication or task repetition, an optimization the fatigued group failed to achieve. Fatigue levels no longer significantly increased during session two in fatigued patients, possibly due to differential effects on energy and cognition from methylphenidate.

The EDA recordings demonstrated lower sympathetic nervous system activity in fatigue patients compared to non-fatigue patients at rest and compared to HC during Stroop task performance (Fig. 7A). EDA normally increases with increased cognitive load, and electrical stimulation of prefrontal cortex can elicit sympathetic cardiovascular responses [34]. Indeed, we observed that increased prefrontal cortex activity was positively correlated to task-related EDL in the non-fatigue, but not in the fatigue group. This suggests dysregulation of the sympathetic nervous system in the fatigue patients.

The diathesis-stress model of psychopathology suggests that vulnerable individuals (due to genetic or environmental factors) that are exposed to stressful life-events may develop psychiatric illness [59]. In our cohort, the life-threatening disease and aHSCT side-effects could constitute such a stressful event, triggering a syndrome of long-lasting fatigue and cognitive dysfunction, paired with structural and functional alterations in the CNS of vulnerable individuals. Indeed, the observed negative correlation between cerebrospinal fluid IL8 levels and prefrontal cortex activity during the Stroop task, in fatigued patients, may be considered as an example of vulnerability to environmental insults.

The limited sample size and cross-sectional design prevents this study from establishing cause-and-effect. Further, while the age difference between patients and HC was mitigated by statistical adjustment, it may still have influenced paradigm performance and cerebral blood flow comparisons between these groups. To test the hypotheses generated by this work, we propose longitudinal studies, beginning before transplant, using matched control groups.

In summary, this study demonstrated a reduced prefrontal cortex and sympathetic nervous system activation during cognitive challenges in patients with fatigue after aHSCT, compared to both non-fatigued patients and healthy controls. Further, the fatigued patients’ weaker responses to methylphenidate suggested a less responsive dopaminergic or noradrenergic system.

## Supplementary information

Supplemental material

## Data Availability

The code used to generate the results is available upon request to the corresponding author.

## References

[1] Hahn T, McCarthy PL, Hassebroek A, Bredeson C, Gajewski JL, Hale GA, et al. Significant improvement in survival after allogeneic hematopoietic cell transplantation during a period of significantly increased use, older recipient age, and use of unrelated donors. J Clin Oncol. 2013;31:2437–49. 23715573 10.1200/JCO.2012.46.6193 PMC3691359

[2] Bower JE, Ganz PA, Desmond KA, Bernaards C, Rowland JH, Meyerowitz BE, et al. Fatigue in long-term breast carcinoma survivors: a longitudinal investigation. Cancer. 2006;106:751–8. 16400678 10.1002/cncr.21671

[3] Esser P, Kuba K, Mehnert A, Schwinn A, Schirmer L, Schulz-Kindermann F, et al. Investigating the temporal course, relevance and risk factors of fatigue over 5 years: a prospective study among patients receiving allogeneic HSCT. Bone Marrow Transpl. 2017. 10.1038/bmt.2016.344. 10.1038/bmt.2016.344 28112750

[4] Mosher CE, Redd WH, Rini CM, Burkhalter JE, DuHamel KN. Physical, psychological, and social sequelae following hematopoietic stem cell transplantation: a review of the literature. Psychooncology. 2009;18:113–27. 18677717 10.1002/pon.1399 PMC3618954

[5] Syrjala KL, Artherholt SB, Kurland BF, Langer SL, Roth-Roemer S, Elrod JB, et al. Prospective neurocognitive function over 5 years after allogeneic hematopoietic cell transplantation for cancer survivors compared with matched controls at 5 years. J Clin Oncol. 2011;29:2397–404. 21537032 10.1200/JCO.2010.33.9119 PMC3107754

[6] Tichelli A, Gerull S, Holbro A, Buser A, Nair G, Medinger M, et al. Inability to work and need for disability pension among long-term survivors of hematopoietic stem cell transplantation. Bone Marrow Transpl. 2017;52:1436–42. 10.1038/bmt.2017.115 28650451

[7] Dietrich J. Neurotoxicity of cancer therapies. Contin Minneap Minn. 2020;26:1646–72. 10.1212/CON.0000000000000943 33273176

[8] Correa DD, Wang Y, West JD, Peck KK, Root JC, Baser RE, et al. Prospective assessment of white matter integrity in adult stem cell transplant recipients. Brain Imaging Behav. 2016;10:486–96. 26153467 10.1007/s11682-015-9423-3 PMC4706509

[9] Correa DD, Root JC, Baser R, Moore D, Peck KK, Lis E, et al. A prospective evaluation of changes in brain structure and cognitive functions in adult stem cell transplant recipients. Brain Imaging Behav. 2013;7:478–90. 23329358 10.1007/s11682-013-9221-8 PMC5536351

[10] Vardy J, Wefel JS, Ahles T, Tannock IF, Schagen SB. Cancer and cancer-therapy related cognitive dysfunction: an international perspective from the Venice cognitive workshop. Ann Oncol. 2008;19:623–9. 17974553 10.1093/annonc/mdm500

[11] Kelly DL, Buchbinder D, Duarte RF, Auletta JJ, Bhatt N, Byrne M, et al. Neurocognitive dysfunction in hematopoietic cell transplant recipients: expert review from the late effects and quality of life working committee of the center for international blood and marrow transplant research and complications and quality of life working party of the european society for blood and marrow transplantation. Biol Blood Marrow Transpl. 2018;24:228–41. 10.1016/j.bbmt.2017.09.004 PMC5768142 28939455

[12] Bernstein LJ, McCreath GA, Komeylian Z, Rich JB. Cognitive impairment in breast cancer survivors treated with chemotherapy depends on control group type and cognitive domains assessed: A multilevel meta-analysis. Neurosci Biobehav Rev. 2017;83:417–28. 29092778 10.1016/j.neubiorev.2017.10.028

[13] Diamond A. Executive functions. Annu Rev Psychol. 2013;64:135–68. 23020641 10.1146/annurev-psych-113011-143750 PMC4084861

[14] Alvarez JA, Emory E. Executive function and the frontal lobes: a meta-analytic review. Neuropsychol Rev. 2006;16:17–42. 16794878 10.1007/s11065-006-9002-x

[15] Ott T, Nieder A. Dopamine and cognitive control in prefrontal cortex. Trends Cogn Sci. 2019;23:213–34. 30711326 10.1016/j.tics.2018.12.006

[16] Berridge CW, Waterhouse BD. The locus coeruleus-noradrenergic system: modulation of behavioral state and state-dependent cognitive processes. Brain Res Brain Res Rev. 2003;42:33–84. 12668290 10.1016/s0165-0173(03)00143-7

[17] Fang C, Lv L, Mao S, Dong H, Liu B. Cognition deficits in Parkinson’s disease: mechanisms and treatment. Park Dis. 2020;2020:2076942. 10.1155/2020/2076942 PMC7128056 32269747

[18] Arnsten AF. The emerging neurobiology of attention deficit hyperactivity disorder: the key role of the prefrontal association cortex. J Pediatr. 2009;154:I–S43. 20596295 10.1016/j.jpeds.2009.01.018 PMC2894421

[19] Arnsten AF, Girgis RR, Gray DL, Mailman RB. Novel dopamine therapeutics for cognitive deficits in schizophrenia. Biol Psychiatry. 2017;81:67–77. 26946382 10.1016/j.biopsych.2015.12.028 PMC4949134

[20] Xing B, Li YC, Gao WJ. Norepinephrine versus dopamine and their interaction in modulating synaptic function in the prefrontal cortex. Brain Res. 2016;1641:217–33. 26790349 10.1016/j.brainres.2016.01.005 PMC4879059

[21] Centeno C, Rojí R, Portela MA, Santiago AD, Cuervo MA, Ramos D, et al. Improved cancer-related fatigue in a randomised clinical trial: methylphenidate no better than placebo. BMJ Support Palliat Care. 2020. 10.1136/bmjspcare-2020-002454. 10.1136/bmjspcare-2020-002454 33168668

[22] Karschnia P, Parsons MW, Dietrich J. Pharmacologic management of cognitive impairment induced by cancer therapy. Lancet Oncol. 2019;20:e92–e102. 30723041 10.1016/S1470-2045(18)30938-0

[23] Gong S, Sheng P, Jin H, He H, Qi E, Chen W, et al. Effect of methylphenidate in patients with cancer-related fatigue: a systematic review and meta-analysis. PloS One. 2014;9:e84391. 24416225 10.1371/journal.pone.0084391 PMC3885551

[24] Miladi N, Dossa R, Dogba MJ, Cléophat-Jolicoeur MIF, Gagnon B. Psychostimulants for cancer-related cognitive impairment in adult cancer survivors: a systematic review and meta-analysis. Support Care Cancer J Multinatl Assoc Support Care Cancer. 2019;27:3717–27. 10.1007/s00520-019-04907-w 31250183

[25] Arenth PM, Ricker JH, Schultheis MT. Applications of functional near-infrared spectroscopy (fNIRS) to Neurorehabilitation of cognitive disabilities. Clin Neuropsychol. 2007;21:38–57. 17366277 10.1080/13854040600878785

[26] Sakudo A. Near-infrared spectroscopy for medical applications: current status and future perspectives. Clin Chim Acta. 2016;455:181–8. 26877058 10.1016/j.cca.2016.02.009

[27] Scarapicchia V, Brown C, Mayo C, Gawryluk JR. Functional magnetic resonance imaging and functional near-infrared spectroscopy: insights from combined recording studies. Front Hum Neurosci. 2017;11:419. 28867998 10.3389/fnhum.2017.00419 PMC5563305

[28] Woodard JL, Sugarman MA. Functional Magnetic resonance imaging in aging and dementia: detection of age-related cognitive changes and prediction of cognitive decline. In: Pardon M-C, Bondi MW (eds). Behavioral neurobiology of aging. Berlin, Heidelberg: Springer; 2012, pp 113–36. 10.1007/7854_2011_159 21922397

[29] Maruishi M, Miyatani M, Nakao T, Muranaka H. Compensatory cortical activation during performance of an attention task by patients with diffuse axonal injury: a functional magnetic resonance imaging study. J Neurol Neurosurg Psychiatry. 2007;78:168–73. 16952916 10.1136/jnnp.2006.097345 PMC2077668

[30] Staffen W, Mair A, Zauner H, Unterrainer J, Niederhofer H, Kutzelnigg A, et al. Cognitive function and fMRI in patients with multiple sclerosis: evidence for compensatory cortical activation during an attention task. Brain J Neurol. 2002;125:1275–82. 10.1093/brain/awf125 12023316

[31] Critchley HD, Eccles J, Garfinkel SN. Interaction between cognition, emotion, and the autonomic nervous system. Handb Clin Neurol. 2013;117:59–77. 24095116 10.1016/B978-0-444-53491-0.00006-7

[32] Patterson JC, Ungerleider LG, Bandettini PA. Task-independent functional brain activity correlation with skin conductance changes: an fMRI study. Neuroimage. 2002;17:1797–806. 12498753 10.1006/nimg.2002.1306

[33] Critchley HD. Neural mechanisms of autonomic, affective, and cognitive integration. J Comp Neurol. 2005;493:154–66. 16254997 10.1002/cne.20749

[34] Critchley HD. Electrodermal responses: what happens in the brain. Neuroscientist. 2002;8:132–42. 11954558 10.1177/107385840200800209

[35] Fagundes CP, Murray DM, Hwang BS, Gouin JP, Thayer JF, Sollers JJ, et al. Sympathetic and parasympathetic activity in cancer-related fatigue: more evidence for a physiological substrate in cancer survivors. Psychoneuroendocrinology. 2011;36:1137–47. 21388744 10.1016/j.psyneuen.2011.02.005 PMC3128662

[36] Bower JE. Cancer-related fatigue-mechanisms, risk factors, and treatments. Nat Rev Clin Oncol. 2014;11:597–609. 25113839 10.1038/nrclinonc.2014.127 PMC4664449

[37] Boberg E, Kadri N, Winterling J, Davies LC, Björklund A, Msghina M et al. Mental fatigue after allogeneic hematopoietic stem cell transplantation is associated with cognitive dysfunction, but not central nervous system inflammation. Haematologica. 2020;105:e310–e314. 10.3324/haematol.2019.225326 PMC7271565 31649133

[38] Challman TD, Lipsky JJ. Methylphenidate: its pharmacology and uses. Mayo Clin Proc. 2000;75:711–21. 10907387 10.4065/75.7.711

[39] Johansson B, Starmark A, Berglund P, Rödholm M, Rönnbäck L. A self-assessment questionnaire for mental fatigue and related symptoms after neurological disorders and injuries. Brain Inj. 2010;24:2–12. 20001478 10.3109/02699050903452961

[40] Veale JF. Edinburgh handedness inventory - short form: a revised version based on confirmatory factor analysis. Laterality. 2014;19:164–77. 23659650 10.1080/1357650X.2013.783045

[41] Stroop JR. Studies of interference in serial verbal reactions. J Exp Psychol. 1935;18:643–62.

[42] Whelan R. Effective analysis of reaction time data. Psychol Rec. 2008;58:475–82.

[43] Bradley MM, Lang PJ. International affective picture system (IAPS): affective ratings of pictures and instruction manual. Technical Report A-8. Gainesville, FL: University of Florida; 2008.

[44] Boucsein W, Fowles DC, Grimnes S, Ben-Shakhar G, Roth WT, Dawson ME, et al. Publication recommendations for electrodermal measurements. Psychophysiology. 2012;49:1017–34. 22680988 10.1111/j.1469-8986.2012.01384.x

[45] Greco A, Valenza G, Lanata A, Scilingo EP, Citi L. cvxEDA: a convex optimization approach to electrodermal activity processing. IEEE Trans Biomed Eng. 2016;63:797–804. 26336110 10.1109/TBME.2015.2474131

[46] Menning S, de Ruiter MB, Veltman DJ, Boogerd W, Oldenburg HS, Reneman L, et al. Changes in brain activation in breast cancer patients depend on cognitive domain and treatment type. PLoS One. 2017;12:e0171724. 28267750 10.1371/journal.pone.0171724 PMC5340346

[47] Silverman DH, Dy CJ, Castellon SA, Lai J, Pio BS, Abraham L, et al. Altered frontocortical, cerebellar, and basal ganglia activity in adjuvant-treated breast cancer survivors 5-10 years after chemotherapy. Breast Cancer Res Treat. 2007;103:303–11. 17009108 10.1007/s10549-006-9380-z

[48] Ferguson RJ, McDonald BC, Saykin AJ, Ahles TA. Brain structure and function differences in monozygotic twins: possible effects of breast cancer chemotherapy. J Clin Oncol J Am Soc Clin Oncol. 2007;25:3866–70. 10.1200/JCO.2007.10.8639 PMC3329758 17761972

[49] McDonald BC, Conroy SK, Ahles TA, West JD, Saykin AJ. Alterations in brain activation during working memory processing associated with breast cancer and treatment: a prospective functional magnetic resonance imaging study. J Clin Oncol. 2012;30:2500–8. 22665542 10.1200/JCO.2011.38.5674 PMC3397784

[50] Yennu A, Tian F, Smith-Osborne A, J Gatchel R, Woon FL, Liu H. Prefrontal responses to Stroop tasks in subjects with post-traumatic stress disorder assessed by functional near infrared spectroscopy. Sci Rep. 2016;6:30157. 27452397 10.1038/srep30157 PMC4995363

[51] Bernstein LJ, Edelstein K, Sharma A, Alain C. Chemo-brain: an activation likelihood estimation meta-analysis of functional magnetic resonance imaging studies. Neurosci Biobehav Rev. 2021;130:314–25. 34454915 10.1016/j.neubiorev.2021.08.024

[52] Ochsner KN, Bunge SA, Gross JJ, Gabrieli JD. Rethinking feelings: an FMRI study of the cognitive regulation of emotion. J Cogn Neurosci. 2002;14:1215–29. 12495527 10.1162/089892902760807212

[53] Wager TD, Davidson ML, Hughes BL, Lindquist MA, Ochsner KN. Prefrontal-subcortical pathways mediating successful emotion regulation. Neuron. 2008;59:1037–50. 18817740 10.1016/j.neuron.2008.09.006 PMC2742320

[54] Yanagisawa H, Dan I, Tsuzuki D, Kato M, Okamoto M, Kyutoku Y, et al. Acute moderate exercise elicits increased dorsolateral prefrontal activation and improves cognitive performance with Stroop test. Neuroimage. 2010;50:1702–10. 20006719 10.1016/j.neuroimage.2009.12.023

[55] Fan J, Flombaum JI, McCandliss BD, Thomas KM, Posner MI. Cognitive and brain consequences of conflict. Neuroimage. 2003;18:42–57. 12507442 10.1006/nimg.2002.1319

[56] Mead LA, Mayer AR, Bobholz JA, Woodley SJ, Cunningham JM, Hammeke TA, et al. Neural basis of the Stroop interference task: response competition or selective attention? J Int Neuropsychol Soc. 2002;8:735–42. 12240737 10.1017/s1355617702860015

[57] Kawakubo Y, Yanagi M, Tsujii N, Shirakawa O. Repetition of verbal fluency task attenuates the hemodynamic activation in the left prefrontal cortex: enhancing the clinical usefulness of near-infrared spectroscopy. PLoS One. 2018;13:e0193994. 29561889 10.1371/journal.pone.0193994 PMC5862477

[58] Linssen AM, Sambeth A, Vuurman EF, Riedel WJ. Cognitive effects of methylphenidate in healthy volunteers: a review of single dose studies. Int J Neuropsychopharmacol. 2014;17:961–77. 24423151 10.1017/S1461145713001594

[59] Broerman R. Diathesis-stress model. In: Zeigler-Hill V, Shackelford TK (eds). Encyclopedia of personality and individual differences. Cham: Springer International Publishing; Cham, 2017, pp 1–3.

