# Correction: Reduced prefrontal cortex and sympathetic nervous system activity correlate with fatigue after aHSCT

**DOI:** 10.1038/s41409-025-02543-z

**Published:** 2025-03-11

**Authors:** Erik Boberg, Ellen Iacobaeus, Myrto Sklivanioti Greenfield, Yanlu Wang, Mussie Msghina, Katarina Le Blanc

**Affiliations:** 1 Department of Laboratory Medicine, Karolinska Institutet, Stockholm, Sweden; 2 Department of Haematology, Karolinska University Hospital, Stockholm, Sweden; 3 Department of Clinical Neuroscience, Karolinska Institutet, Stockholm, Sweden; 4 Medical Radiation Physics and Nuclear Medicine, Karolinska University Hospital, Stockholm, Sweden; 5 Radiology, Clinical Sciences, Intervention and Technology, Karolinska Institutet, Stockholm, Sweden; 6 School of Medical Sciences, Örebro University, Örebro, Sweden; 7 Department of Cellular therapy and Allogeneic Stem Cell Transplantation, Karolinska University Hospital, Stockholm, Sweden

Correction to: *Bone Marrow Transplantation* 10.1038/s41409-021-01539-9, published online 04 December 2021

In this article in Table 1, in the section that lists the underlying diseases of the included patients, one patient in each group is not listed. One patient in the fatigued group that had MDS/AML and one patient in the non-fatigued group that had Myelofibrosis/AML. We would like to stress that these two patients are not missing from any other part of the paper, and that the accidental omission from the list of diagnoses does not in any way alter the conclusions drawn. The p-value of 0.11 for the Fisher’s exact test comparing the distribution of underlying haematological disorders between the study groups is correct, as all study participants were included in that calculation.

**Table 1:** Characteristics of the study population.

	Fatigued (*n* = 12)	Non-fatigued (*n* = 12)	*P*
Time from transplant to fNIRS testing (months): median (range)	46 (16–66)	47.5 (22–68)	0.68^t^
Donor type: *n* (%)			
MUD	1 (8)	1 (8)	1^†^
SIB	4 (33)	4 (33)	
Haplo	7 (58)	7 (58)	
Underlying disease: *n* (%)			
AML	4 (33)	9 (75)	0.11^†^
CML	0 (0)	0 (0)	
MDS	1 (8)	2 (17)	
MDS/AML	1 (8)	0 (0)	
PMF	3 (25)	0 (0)	
PMF/AML	0 (0)	1 (8)	
Myeloma	1 (8)	0 (0)	
CLL	1 (8)	0 (0)	
Sickle cell anemia	1 (8)	0 (0)	
Conditioning regimen drugs: *n* (%)			
Busulfan	8 (67)	9 (75)	1^†^
Cyclophosphamide	3 (25)	2 (17)	1^†^
Fludarabine	9 (75)	10 (83)	1^†^
Treosulfan	4 (33)	3 (25)	1^†^
Thiotepa	2 (17)	0 (0)	0.48^†^
Immune reconstitution			
Days from transplant to neutrophils > 0.5 × 10^9^/L (days): median (range)	16 (12–28)	17 (11–19)	0.93^#^
CMV and EBV: *n* (%)			
CMV mismatch	4 (33)	2 (17)	0.64^†^
EBV mismatch	0 (0)	1 (8)	1^†^
CMV reactivation^a^	6 (50)	4 (33)	0.68^†^
EBV reactivation^b^	2 (17)	1 (8)	1^†^
GvHD prophylaxis: *n* (%)			
ATG	8 (67)	7 (58)	1^†^
Ciclosporin	8 (66)	9 (75)	0.68^†^
Tacrolimus	3 (25)	1 (8)	
Tacrolimus + Sirolimus	1 (8)	2 (17)	
GvHD: *n* (%)			
Acute^c^	9 (75)	7 (58)	0.67^†^
Chronic^d^	5 (42)	4 (33)	1^†^
Regular opioid, antidepressive or anxiolytic medication: *n* (%)			
SSRI	2 (17)	0 (0)	0.48^†^
Opioids	2 (17)	0 (0)	0.48^†^

^t^Students t-test (continuous data with parametric distribution according to the Shapiro-Wilk test) ^#^Wilcoxon Rank Sum Test (continuous data with non-parametric distribution according to the Shapiro-Wilk test), ^†^Fisher’s exact test (categorical data).

*MUD* matched unrelated donor, *SIB* matched sibling donor, *Haplo* Haploidentical donor, *AML* acute myeloid leukemia, *CML* chronic myeliod leukemia, *MDS* myelodysplastic syndrome, *PMF* primary myelofibrosis, *CLL* chronic lymethylphenidateocytic leukemia, *CMV* cytomegalovirus, *EBV* Epstein Barr virus, *GvHD* Graft versus Host Disease, *ATG* anti thymocyte globulin, *SSRI* selective serotonin reuptake inhibitor.

^a^Defined as CMV DNA > 1000 copies/ml.

^b^Defined as EBV DNA > 1000 copies/ml.

^c^Previous acute GvHD requiring steroid treatment.

^d^Previous/current cGvHD.

**Corrected Table 1**

In the supplemental material, supplemental Table 1 we list inclusion criteria for the study. There, we have written “Underwent allogeneic HSCT for hematologic malignancy ≥12 months and ≤5 years ago”. Since we also allowed inclusion of patients transplanted for benign disorders (in fact, one of the included patients was transplanted for sickle cell anemia) the inclusion criterion should say: “Underwent allogeneic HSCT for hematologic disorder ≥12months and ≤5 years ago”.

The original article has been corrected.

## Supplementary information

Supplemental material Corrected

