# Supplementary material for: Reduced prefrontal cortex and sympathetic nervous system activity correlate with fatigue after aHSCT
Source: Bone Marrow Transplant. 2021 Dec 4;57(3):360–9. doi: 10.1038/s41409-021-01539-9 (PMC8907068; doi:10.1038/s41409-021-01539-9)
Supplement: Supplementary file 1 — Supplemental material [file 41409_2021_1539_MOESM1_ESM.pdf]

Supplemental material

Supplemental methods

Study blinding

A single dose of methylphenidate was administered to all participants before session 2. To reduce the placebo effect, the subjects were informed that they would receive either methylphenidate or placebo. Thus, the subjects were blinded to the treatment that they received, but the researchers were not, i.e., the study was single-blinded.

Detailed description of the cognitive tests

The Stroop task

The colour word Stroop task (referred to as "the Stroop task" from now on) tests the subjects ability to select the appropriate response when presented with conflicting stimuli, also known as cognitive interference[1]. The Stroop task is exemplified in Figure 2a. The subjects were presented with two words for colour names above each other. The top word was always written in black letters while the bottom word was written in different colours (red, green, blue, yellow or black). The participants were instructed to press "1" on a computer keyboard if the meaning of the top word corresponded to the colour of the text of the bottom word, and if not, to press "2". The individual tasks were either congruent, where the colour of the text of the bottom word and the meaning of the bottom word corresponded, or incongruent, where the colour of the text of the bottom word did not correspond to the meaning of that word (fig 2a). The test was divided into 8 blocks of 15 trials per block, randomized between congruent and incongruent tasks. The proportion of congruent and incongruent tasks was 70/30. Each trial was 2 seconds (s) long, making each block 30s long. There was a 30s rest period between each block. Reaction time and proportion of correct answers were registered. Trials with reaction-time of <200ms and trials in which no response was recorded before the next trial commenced (>2000ms reaction time) were removed from the analysis[2]. Very brief reaction times cannot be considered to represent actual task responses, due to physiological limitations[2]. Furthermore, due to the skewness of reaction time data, the median reaction time of each individual was used as outcome[2]. Before data registration began, all subjects were allowed to repeatedly perform Stroop trials until they felt comfortable with the paradigm. Among the HC, 25 subjects had completed the Stroop task and their data was compared to the

patient data. Since the HC had only performed four blocks of Stroop trials, we selected the first four blocks from the two sessions in our comparison to the HC data.

The verbal fluency task

In the verbal fluency task, the subjects were presented with five letters (a, i, o, e and u), one at a time. Each letter was presented for 20s and the task was to generate as many words as possible beginning with that particular letter, within that time frame. Names of persons, countries, cities, etc were not allowed. Before the task phase, the test started with a 20s rest phase, followed by 20s where all five letters were displayed and the subject was instructed to repeat the letters out loud (the vocalization phase), to activate the speech centres in the CNS (fig 5a). This served as the baseline for the fNIRS and EDA registrations. Only one block of verbal fluency task was administered per session[3]. Since the verbal fluency task was not included in the study by Sklivanioti et al [REF], we were not able to compare our results to HC.

The emotion regulation task

During the emotion regulation task, participants were presented with five images of negative emotional valence in sequence[4]. The images were presented six times, with a 30s rest phase between each block (fig 6a). During the rest phases the participants were randomly instructed on screen to either passively observe the next set of images (induction) or actively attempt to reduce the intensity of the negative emotions that were provoked by them using reappraisal (i.e to interpret the images in a more positive way). As a performance metric, we used the self-reported emotional intensity on a numeric rating scale (NRS). The relative difference in intensity (I) between the induction (ind) and regulation (reg) phases was then calculated, i.e. $(I_{ind} - I_{reg}) / I_{ind}$. Among the HC, 27 subjects had completed the negative emotions task and their data was compared to the patient data.

fNIRS registration and preprocessing

fNIRS registration was performed using a Biopac fNIR model 1100-V3.2A (fNIR Devices LLC, Potomac, MD, USA) with a 16-channel sensor. The headband was applied so that the

detectors were centred on the forehead, attempting to remove any hair under the detectors to optimize signal quality (suppl fig 1a). To reduce light pollution, the registration was performed in a dimly lit room and, when necessary, a cap was placed over the detector for further noise reduction. After applying the headband, the initial gain and LED drive current parameters were adjusted to obtain a stable signal from both wavelengths (730 and 850nm) for all channels above the detection threshold without ceiling effects. fNIRS data was captured using COBI Studio software (Biopac systems inc, Goleta, CA, USA).

Raw light intensity output was converted to levels of oxygenated and deoxygenated hemogobin (HbO and HbR, respectively) using the built-in models in the COBI studio software. Due to the low signal-to-noise ratio of HbR, only HbO data was included in this study. Physiological noise (i.e. artifacts from respiration and cardiac pulsation) was removed using two band-stop filters (0.12-0.25 and 0.7-2.0Hz). Data from the Stroop and emotion regulation tasks were detrended using a high-pass filter based on a discrete cosine transform set with the cut-off period set to 128s. The verbal fluency task data was not detrended since that task was sufficiently short for baseline drift to not be a major concern. Motion artefact correction was not applied, since the participants remained stationary in a chair for the duration of testing. Using generalized linear modelling (GLM) the data from each channel was fitted to the hemodynamic response function and the resulting beta coefficients were used for between-group comparisons. Data analysis was conducted using Matlab version 2020b (Mathworks inc, Natick, MA, USA), with the package SPM for fNIRS toolbox[5].

EDA registration and preprocessing

The EDA signal was recorded using the MP150 data acquisition and analysis system (Biopac systems inc, Goleta, CA, USA) through silver-silver chloride (Ag-AgCl) electrodes attached to dig 2 and 3 of the left hand (suppl fig 1e). Data was captured using the Acqknowledge software version 4.3 (Biopac systems inc, Goleta, CA, USA).

A 5th-order low-pass Butterworth filter with cut-off frequency at 1Hz, as well as median smoothing with a smoothing window equal to the frequency of the sample (8Hz), were applied to the raw EDA signal, to eliminate artefacts and high-frequency noise. Next, the

EDA signal was decomposed into three components (the tonic signal, phasic signal and white Gaussian noise), using a convex optimization approach[6] (suppl fig 1f). Fixed values of $\tau 1 = 0.7s$, $\tau 0 = 2s$, δ knot = 10, $\alpha = 0.0008$ and $\gamma = 0.01$ were used.

The tonic signal, referred to as the electrodermal conductance level (EDL), reflects overall degree of arousal. The phasic component, referred to as electrodermal conductance responses (EDR), can reflect either short-term responses to external stimuli if they are observed within 1-3s after stimulus onset, or overall degree of arousal if they are observed during rest (Non-specific EDR, NS-EDR). In this study, three EDA features were calculated from the tonic and phasic EDA components: 1) The difference in EDL amplitude between the final 10s of each rest phase and the 10s with highest amplitudes during the following task phase. 2) The mean EDR amplitude during each task phase. 3) The mean NS EDR amplitude during each rest phase. The EDR:s were automatically detected and measured using the EDA toolbox extension for Matlab (available online at https://github.com/mateusjoffily/EDA/wiki).

Statistical analysis

Paradigm performance and numerical rating scales

For analysis of behavioural performance and numerical rating scales, normality was first tested using the Shapiro Wilks test. If the data was considered normal (i.e. Shapiro Wilks $p > 0.05$), paired and unpaired t-tests were used. Otherwise, the Wilcoxon rank-sum and Wilcoxon signed-rank tests respectively were used. The comparisons to HC were age-adjusted using linear models with the performance metric as dependent variable, and study group (1: Fatigue, 0: Non-Fatigue, -1: HC) and age (in years) as independent variables.

fNIRS data analysis

The beta coefficients derived from the fNIRS GLM were individually compared for each of the 16 channels. A channel was considered activated if the one-sample t-test was significant (the coefficients were significantly different from 0). Further, the beta coefficients were compared between patients with and without fatigue using unpaired t-tests and between before and after MPH using paired t-tests. Linear models with channel beta coefficient as dependent variable and study group (1: Fatigue, 0: Non-Fatigue, -1: HC) and age (in years) as independent variables were used when comparing patients and HC. Pearson correlation was

used to determine the relationship between changes in channel activation and changes in task performance.

EDA data analysis

We assumed a logarithmic habituation of the EDA signal over time and analysed the EDA data using linear mixed effects modelling. For comparisons between fatigued and non-fatigued patients we used EDA feature as dependent variable; patient group (1: Fatigue, 0: Non-fatigue), session (before/after MPH) and log of block number as independent variables; and subject as the random effect factor. The interactions between patient group and session, patient group and block number, and session and block number were included. For comparison to HC, each session was analysed independently with EDA feature as dependent variable; study group (1: Fatigue, 0: Non-fatigue, -1: HC), log of block number and age as independent variables; and subject as the random effect factor. The interaction between study group and block number was included.

Significant interactions were explored using reduced models. For example, a significant Patient group - Session interaction was explored by separately fitting a model without the independent variable patient group, to the fatigue and non-fatigue groups.

Age-adjusted comparisons to healthy controls

Due to the significant difference in age between the healthy controls and the patient groups, all comparisons to healthy controls were age-adjusted by using linear regression and including age as an independent variable in the regression models[7].

Homogeneity of variance

Equality of variance between the study groups was assessed using Levene's test. The variance was homogeneous for all comparisons of paradigm performance between the patient groups. Residual vs fitted plots of the linear models used for age-adjustment between HC and the patient groups occasionally indicated heteroscedasticity. The variances were homogeneous for >95% of the comparisons of fNIRS beta coefficients. Consequently, we chose to perform all those comparisons with a t-test. Applying a Levene's test to the residuals from the linear mixed effects models used to analyse the EDA data indicated heterogeneity of variance. However, linear mixed effects models provide an intuitive way to describe the EDA data in

this study, and have been demonstrated to be fairly robust to violations of assumptions[8]. Thus, we believe that the benefits of this approach outweigh its disadvantages.

Correlation to immune cell subsets and cytokine levels in cerebrospinal fluid

In an exploratory analysis, the beta coefficients from the fNIRS channels that differed between fatigue and non-fatigue patients were correlated to cytokine concentrations and immune cell subsets measured in our previous study on the same cohort of patients[9]. Multiple comparison correction using the Benjamini-Hochberg method was applied to these comparisons.

Due to the exploratory nature of this study, no further corrections for multiple comparisons were performed.

Supplemental tables

Supplemental table 1: Inclusion and exclusion criteria

**Inclusion criteria**	**Exclusion criteria**
Over 18 years old	History of intracranial infection
Underwent allogeneic HSCT for hematologic disorder ≥12months and ≤5 years ago.	History of intrathecal chemotherapy
	Current active or chronic neurological or psychiatric disorder, such as stroke, inflammatory neurological disease, schizophrenia, severe depression, suicide tendencies, anorexia nervosa, severe mood swings, bipolar disorder or any type of dementia or other degenerative neurological disease.
	Current use of antipsychotic drugs, tricyclic antidepressants (in higher doses used for treating depression), high doses of benzodiazepines (20mg of diazepam daily) or equivalents, high doses of opioids (30mg of morphine or equivalent daily).
	Current or history of substance abuse.
	Currently on a steroid dose equal to 15mg of prednisolone or more.
	MRI showing signs of elevated intracranial pressure or increased risk of herniation following lumbar puncture.
	Increased risk of bleeding (i.e. thrombocytes < 50×10^9/L, Prothrombin Time, International Normalized Ratio > 1.4) complicating lumbar puncture.
	Skin infection at the location for lumbar puncture (above the L3/L4 or L4/L5 lumbar vertebra).

	Magnetic implant, or implanted device that may be affected by the magnetic field inside the MRI machine.
	Treated with total body irradiation as part of conditioning regiment for HSCT.

Inclusion and exclusion criteria used when recruiting the study population. See also the previous publication[9].

Supplemental table 2: Contraindications to treatment with methylphenidate

Hypersensitivity to methylphenidate or any of its excipients
History of glaucoma or pheochromocytoma
Currently treated with monoaminooxidase inhibitors or withdrew from monoaminooxidase inhibitors within 14 days
Hyperthyroidism or thyrotoxicosis
Severe hypertension, heart failure, arterial occlusive disorder, angina pectoris, hemodynamicly significant congenital heart disease, cardiomyopathy, myocardial infarction, potentially life threatening arrhythmias, channelopathys
Cerebrovascular disorders, erebral aneurysms, cerebral vasculitis, stroke

Supplemental table 3: Comparative characteristics for the patient and healthy control cohorts

	**Fatigued (n=12)**	**Non-fatigued (n=12)**	**Healthy controls (n=27)**	**P**
**Age:** mean (range)	50.8 (24-67)	57.9 (23-75)	31 (22-55)	<0.0001[†]
**Sex (male):** n (%)	4 (33)	7 (58)	12 (46)	0.50[#]
**Highest education level:** n (%)				
Elementary school	0 (0)	1 (8)	0 (5)	
High school	5 (42)	2 (17)	3 (12)	0.09[#]
College/University	7 (58)	9 (75)	23 (88)	

[†] P-value calculated using the Kruskal Wallis test. Post hoc Dunn's test with Benjamini-Hochberg adjustment for multiple comparisons: Fatigued vs Healthy controls p=0.0054, Non-fatigued vs Healthy controls p<0.0001, Fatigued vs Non-fatigued p=0.24

[#] P-value calculated using Fisher's exact test.

Supplemental figures

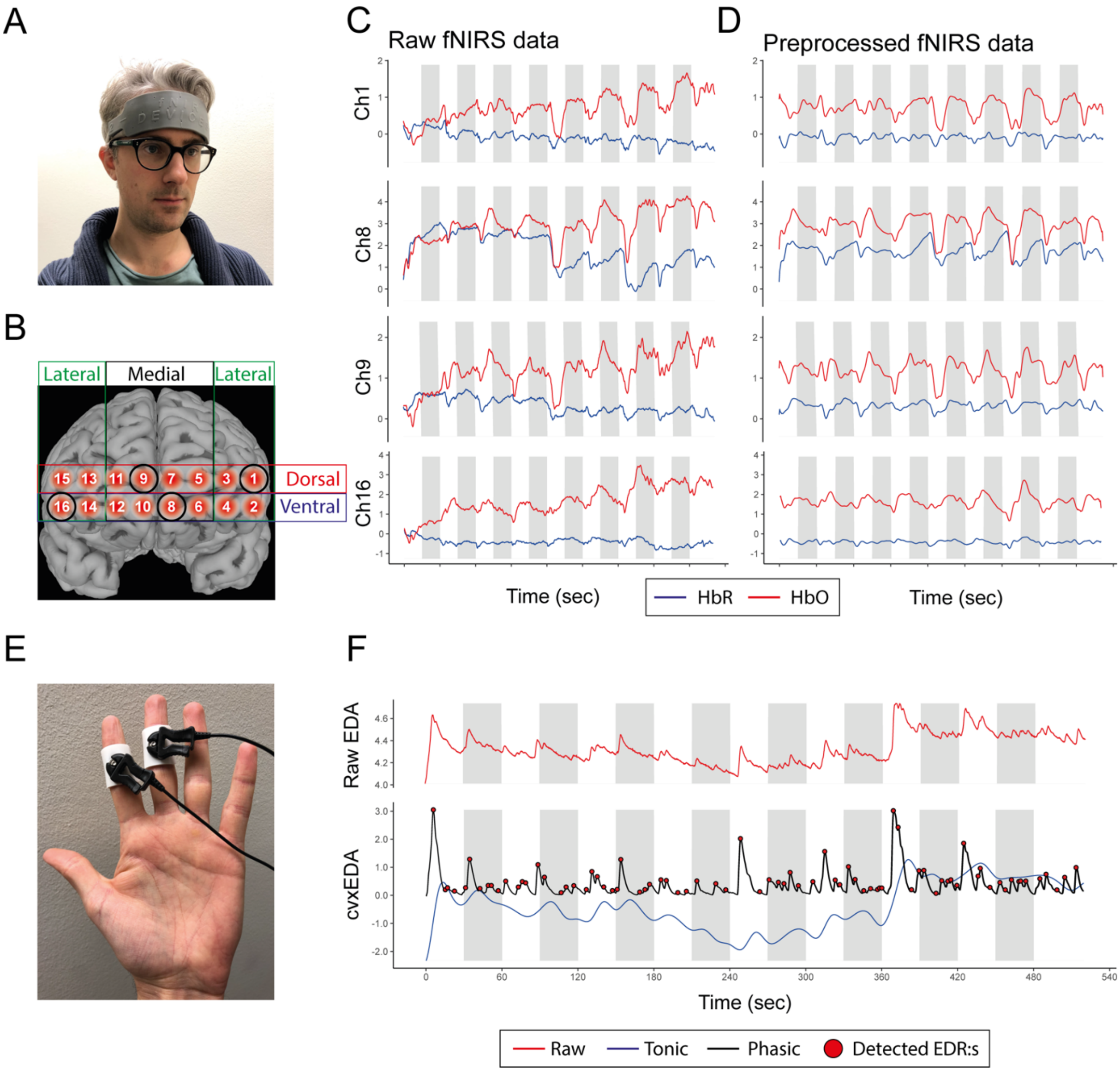

Supplemental figure 1: fNIRS and EDA analysis. A) The 16-channel fNIRS headband was centred on each subject's forehead. The person in the picture is the author E.B. B) The approximate anatomical location of each of the 16 channels. C-D) Examples of fNIRS signals from the Stroop task before and after pre-processing (band-stop filtering and detrending). The grey areas represent each task block and the white areas between represent resting phases. E) Placement of Ag-AgCl electrodes for EDA registration on the left middle phalanges of dig 2 and 3. F) Example of raw and pre-processed (low-pass filtering, smoothing, and decomposing) EDA signal from the Stroop task. The red line represents the raw data, the blue line the tonic component and the black line the phasic component. Red dots represent automatically detected EDRs. fNIRS: functional Near-Infra-Red Spectroscopy, EDA: Electrodermal Activity, EDRs: Electrodermal Responses, Ch: Channel, HbR: Deoxygenated Haemoglobin, HbO: Oxygenated Haemoglobin, cvxEDA: convex optimization EDA.

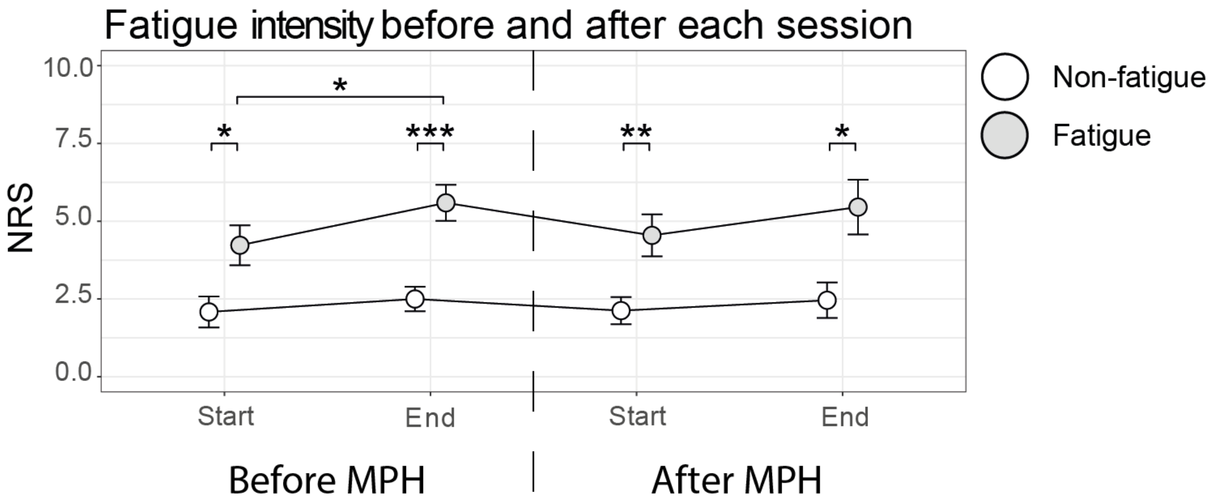

Supplemental figure 2: Fatigue levels before and after each session. Fatigue was measured using a numeric rating scale before and after each session. As expected, the fatigued patients rated a higher degree of fatigue on all measurements. The level of fatigue increased significantly in the fatigued patients during the session before MPH, but not during the session after MPH. In the non-fatigued patients, the level of fatigue remained constantly low throughout the procedure. MPH: Methylphenidate

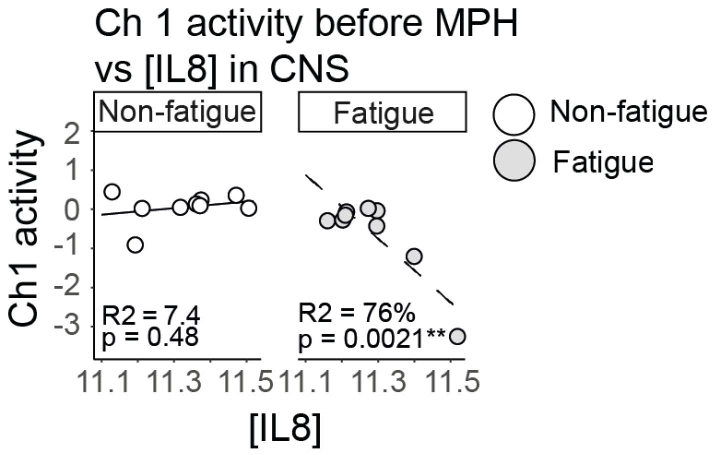

Supplemental figure 3: Ch 1 activity is correlated to IL8 concentration in fatigued, but not non-fatigued patients. Since there was a significant difference in activity between fatigued and non-fatigued patients for ch 1 before MPH, we correlated that activity to IL8 concentration in the CSF, that was measured in a previous study on this cohort[7]. IL8 concentration was negatively correlated to ch 1 activity in fatigued, but not in non-fatigued patients. Ch: Channel, IL: Interleukin, MPH: Methylphenidate, CSF: Cerebrospinal Fluid

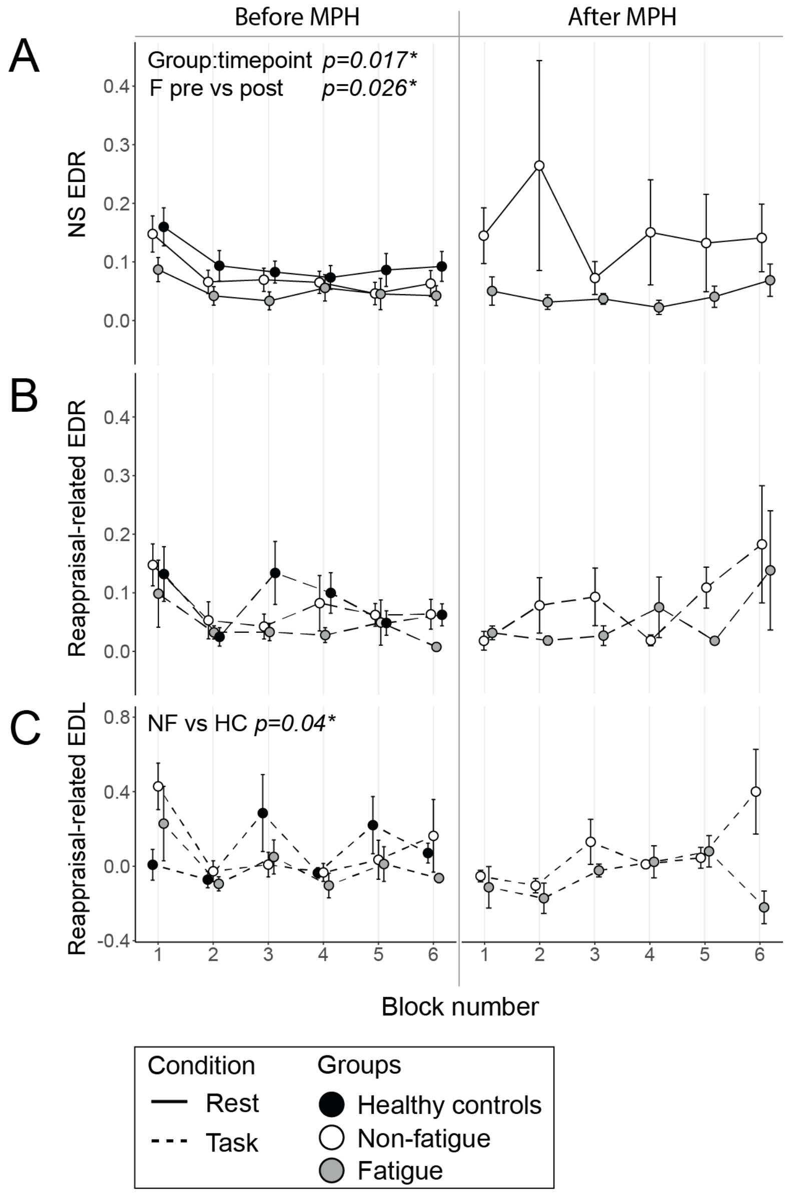

Supplemental figure 4: The negative emotion task – EDA. The fatigued and non-fatigued groups were compared to each other and to the HC using linear mixed models. The comparisons to HC were age-adjusted. The p-values shown are derived from those models. Refer to the materials and methods section for details. A) The fatigued group significantly decreased in NS EDR after receiving MPH, but no change was noted for the non-fatigued group (group:timepoint interaction p=0.017*, before vs after MPH in fatigued p=0.026*). B) No differences between the groups were seen for the reappraisal related EDR. C) Before MPH, the non-fatigued group had significantly higher reappraisal related EDL increase compared to HC. EDA: Electrodermal Activity, EDR: Electrodermal Response, EDL: Electrodermal Level, HC: Healthy Controls

References

1. Stroop, J. R. Studies of interference in serial verbal reactions. *Journal of Experimental Psychology* **18**, 643–662 (1935).
2. Whelan R. Effective Analysis of Reaction Time Data. *The Psychological Record* **58**, 475–482 (2008).
3. Suto, T., Fukuda, M., Ito, M., Uehara, T. & Mikuni, M. Multichannel near-infrared spectroscopy in depression and schizophrenia: cognitive brain activation study. *Biol Psychiatry* **55**, 501–11 (2004).
4. Lang P. J., B. M. M. *International affective picture system (IAPS): Affective ratings of pictures and instruction manual. Technical Report A-8.* (2008).
5. Tak, S., Uga, M., Flandin, G., Dan, I. & Penny, W. D. Sensor space group analysis for fNIRS data. *J Neurosci Methods* **264**, 103–112 (2016).
6. Greco, A., Valenza, G., Lanata, A., Scilingo, E. P. & Citi, L. cvxEDA: A Convex Optimization Approach to Electrodermal Activity Processing. *IEEE Trans Biomed Eng* **63**, 797–804 (2016).
7. Kahlert, J., Gribsholt, S. B., Gammelager, H., Dekkers, O. M. & Luta, G. Control of confounding in the analysis phase – an overview for clinicians. *Clin Epidemiol* **9**, 195–204 (2017).
8. Schielzeth, H. *et al.* Robustness of linear mixed-effects models to violations of distributional assumptions. *Methods in Ecology and Evolution* **11**, 1141–1152 (2020).
9. Boberg, E. *et al.* Mental fatigue after allogeneic hematopoietic stem cell transplantation is associated with cognitive dysfunction, but not central nervous system inflammation. *Haematologica* haematol.2019.225326 (2019) doi:10.3324/haematol.2019.225326.
